# Mechanoreceptor Stretching in the Superior Tarsal Muscle Triggering Limb and Craniocervical Dystonia: A Report of Two Cases

**DOI:** 10.7759/cureus.112526

**Published:** 2026-07-12

**Authors:** Kiyoshi Matsuo, Ai Kaneko

**Affiliations:** 1 Plastic and Oculoplastic Surgery, Matsuo Plastic and Oculoplastic Surgery Clinic, Hamamatsu, JPN; 2 Plastic and Reconstructive Surgery, Shinshu University School of Medicine, Matsumoto, JPN

**Keywords:** facial grimacing muscle, inferior tarsal muscle, jaw opening muscle, limb dystonia, mechanoreceptors, meige syndrome, neck rotation muscle, rostral locus coeruleus, superior tarsal muscle, tongue thrusting muscle

## Abstract

Voluntary upper eyelid opening stretches mechanoreceptors in the superior tarsal muscle (STM), serving as an analog to a serial muscle spindle, inducing reflex contractions of slow-twitch fibers in the levator palpebrae superioris and facial expression muscles via the mesencephalic trigeminal nucleus and rostral locus coeruleus (LC). Because the LC projects widely to the brainstem and spinal cord, we hypothesized that excessive STM mechanoreceptor stretching could induce dystonic contractions across broader muscle groups. This observational case report describes two unique patients presenting with multifocal and limb dystonia. Diagnostic mechanical testing (eyebrow elevation and cheek depression via taping) and sensory masking (high-frequency focal vibration) were utilized to evaluate the immediate modulation of proprioceptive inputs. Therapeutic interventions included oculoplastic surgical reconstruction with longitudinal follow-up intervals ranging from 1 week to 18 months.

Case 1 involved a 29-year-old man with severe levator aponeurosis disinsertion presenting with task-specific limb flexion dystonia. Mechanical reduction of the STM stretch, initially via taped eyebrow elevation and subsequently through corrective surgery, completely resolved the dystonic movements. Case 2 involved a 38-year-old man who developed left-predominant craniocervical and limb dystonia following lower eyelid surgery, driven by increased reflex contractions of the palpebral orbicularis oculi muscle opposing eyelid opening. Symptoms were significantly alleviated either by facial taping to reduce STM stretching or by applying high-frequency focal vibration to trigeminal nerve branches to mask the abnormal proprioceptive input. Secondary metabolic, drug-induced, and primary genetic etiologies were formally ruled out in both cases. These cases suggest a novel, hypothesis-generating model wherein excessive proprioceptive stimulation from STM mechanoreceptors hyperactivates the rostral LC, potentially exerting downstream tonic influences on spinal and brainstem motor pathways. While these striking clinical outcomes highlight a plausible ocular-proprioceptive trigger for craniocervical and peripheral dystonias, prospective controlled studies are required to validate this pathophysiological mechanism.

## Introduction

The opening of the upper eyelid plays a crucial role in coordinating the movements of the eyelid, eyebrow, and face. This coordinated movement relies on a specialized proprioceptive (position-sensing) feedback loop within the periocular architecture. Voluntary contractions and microsaccades (tiny, involuntary eye movements) [1] of fast-twitch fibers (FTFs) in the levator palpebrae superioris muscle (LPSM; the primary eyelid elevator) and the global layer of the superior rectus muscle (GLSRM; involved in upward gaze) [2] stretch mechanoreceptors embedded within the smooth muscle fibers of the superior tarsal muscle (STM). The STM functions as a mechanical sensor analogous to a serial muscle spindle [3,4]. Via the mesencephalic trigeminal nucleus (MTN; a brainstem nucleus containing primary proprioceptive neurons) and the rostral locus coeruleus (LC; a core noradrenergic modulating center) [5], this peripheral stretch input travels to brainstem motor centers. This pathway reflexively induces phasic and tonic contractions of slow-twitch fibers (STFs) in the LPSM [6], the occipitofrontalis muscle (eyebrow elevator) [7,8], the orbital part of the orbicularis oculi muscle (OOM; outer eyelid squeezing muscle) [8,9], other facial expression muscles [9], and the upper trapezius muscle in the neck [10]. Furthermore, STM mechanoreceptor stretching activates the forebrain through the MTN-LC axis, regulating physiological arousal by increasing prefrontal blood flow and sympathetic activity (e.g., palmar sweating) [11], which concurrently increases microsaccade velocity [1].

Simultaneously, maintaining a stable visual axis requires counteracting contractions and microsaccades of FTFs in the global layer of the inferior rectus muscle (GLIRM; involved in downward gaze) [2]. These lower ocular movements directly oppose those in the LPSM and GLSRM, stretching putative mechanoreceptors within the inferior tarsal muscle (ITM) of the lower eyelid [12]. This ITM stretch, in turn, induces reflex contractions of STFs in the palpebral part of the OOM (inner eyelid closing muscle), essentially adhering both the upper and lower eyelids tightly to the globe during gaze shifts [12].

Individuals who habitually rub or stretch their eyelids due to chronic pollen allergies, atopic dermatitis, or the frequent application of contact lenses and eyedrops commonly experience physical disinsertion (detachment) of the levator aponeurosis and inferior tarsal retractor from the tarsal plates [9,10,12,13]. To compensate for this mechanical slack and maintain adequate eyelid opening post-disinsertion, patients must significantly increase voluntary contractions and microsaccades of FTFs in the LPSM and GLSRM. This compensatory hyperdrive markedly enhances STM mechanoreceptor stretching, leading to heightened reflex contractions of STFs in the LPSM [13], occipitofrontalis muscle [7,8], and orbital OOM [9]. However, these increased STF contractions in the orbital OOM functionally resist upper eyelid opening from the outside. Concurrently, to oppose the hypercontracted GLSRM, patients must also enhance the counteracting contractions and microsaccades of FTFs in the GLIRM, aggravating the stretching of putative mechanoreceptors in the ITM. The resulting increase in reflex contractions of STFs in the palpebral OOM [12] further resists upper eyelid opening from the inside. Together, these external and internal antagonist resistances from the orbital and palpebral OOMs against upper eyelid opening can trigger profound, continuous stretching of the STM mechanoreceptors.

Because the LC sends extensive noradrenergic projections throughout the central nervous system, including the forebrain, brainstem, cerebellum, and spinal cord motor centers [14-18], we formulated a novel working hypothesis: the rostral LC, when pathologically hyperactivated by excessive STM mechanoreceptor stretching, could induce widespread reflex contractions of STFs in broader muscle groups far beyond the periocular and facial regions. To explore this hypothesis, we describe two cases of severe dystonic muscle contractions secondary to pathological STM mechanoreceptor stretching, resulting either from severe aponeurosis disinsertion or from secondary hypercontraction of the palpebral OOM.

This report was previously presented as an oral presentation at the 32nd Research Council Meeting of the Japan Society of Plastic and Reconstructive Surgery on October 19-20, 2023.

## Case presentation

**Figure 1:**
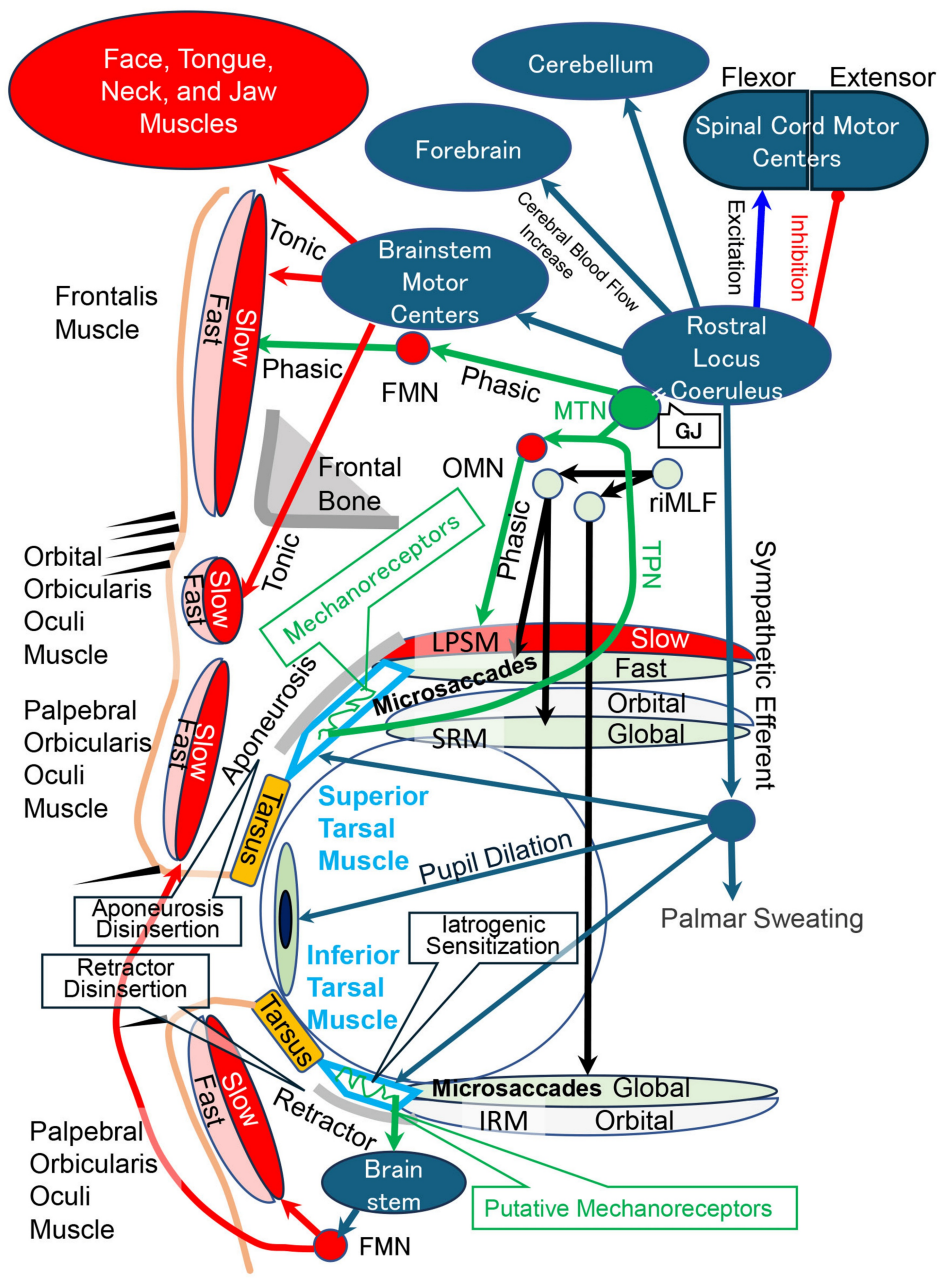
Neuroanatomy of reflex contractions in limb and craniocervical muscles via the rostral locus coeruleus Aponeurosis and retractor disinsertion from the tarsi increases the voluntary contractions and microsaccades of fast-twitch fibers (Fast) in the levator palpebrae superioris muscle (LPSM) and the global layers of the superior rectus muscle (SRM) and inferior rectus muscle (IRM), driven by the activation of the rostral interstitial nucleus of the medial longitudinal fasciculus (riMLF) and the oculomotor nucleus (OMN). These heightened contractions enhance mechanoreceptor stretching in the superior tarsal muscle (STM) and inferior tarsal muscle (ITM). Stretching of putative mechanoreceptors in the ITM reflexively contracts slow-twitch fibers (Slow) in the palpebral orbicularis oculi muscle (OOM) via the brainstem. The rostral locus coeruleus (LC), connected to the mesencephalic trigeminal nucleus (MTN) via gap junctions (GJ), projects extensively to the forebrain (increasing cerebral blood flow), brainstem, cerebellum, and spinal cord. Mechanoreceptor stretching in the STM induces reflexive, phasic contractions of slow-twitch fibers (Slow) in the LPSM and frontalis muscle via the MTN, OMN, and FMN. Concurrently, it drives reflexive, tonic contractions of slow-twitch fibers (Slow) in craniocervical muscles, including the orbital OOM (facial grimacing), tongue-thrusting muscles, and neck rotation muscles, via the MTN, rostral LC, and brainstem motor centers (FMN, hypoglossal nucleus, and accessory nucleus). Additionally, it exerts a tonic excitatory influence on limb flexor muscles and an inhibitory influence on limb extensor muscles via spinal motor pathways, resulting in limb dystonia. Image Credit: Kiyoshi Matsuo and Ai Kaneko created the figure using Microsoft PowerPoint (Microsoft Corporation, Redmond, WA, US)

Figure 1 illustrates the neuroanatomical pathways involved in this pathology. Pathological stretching of the mechanoreceptors in the STM, resulting either from levator aponeurosis disinsertion from the tarsi or from secondary hypercontraction of STFs in the palpebral OOM, due to iatrogenically sensitized mechanoreceptors in the ITM, reflexively triggers dystonic contractions. Via the MTN and the rostral LC, this proprioceptive input stimulates spinal and brainstem motor centers, leading to dystonic contractions in limb flexor and extensor muscles and muscles governing facial grimacing, tongue thrusting, jaw opening, and neck rotation.

To rule out alternative etiologies for this novel presentation, a comprehensive neurological workup was performed by the patients' previous neurologists. Brain magnetic resonance imaging (MRI) revealed no structural lesions in the basal ganglia or brainstem; serum ceruloplasmin and copper levels were within normal limits, ruling out Wilson’s disease; and a detailed pharmacological history confirmed no exposure to neuroleptic agents, excluding drug-induced tardive dystonia.

Written informed consent was obtained from both patients for the publication of these case reports and any accompanying images. The patient in Case 2 explicitly granted permission for publication without any obscuring modifications to the photographic or video materials.

Case 1

**Figure 2:**
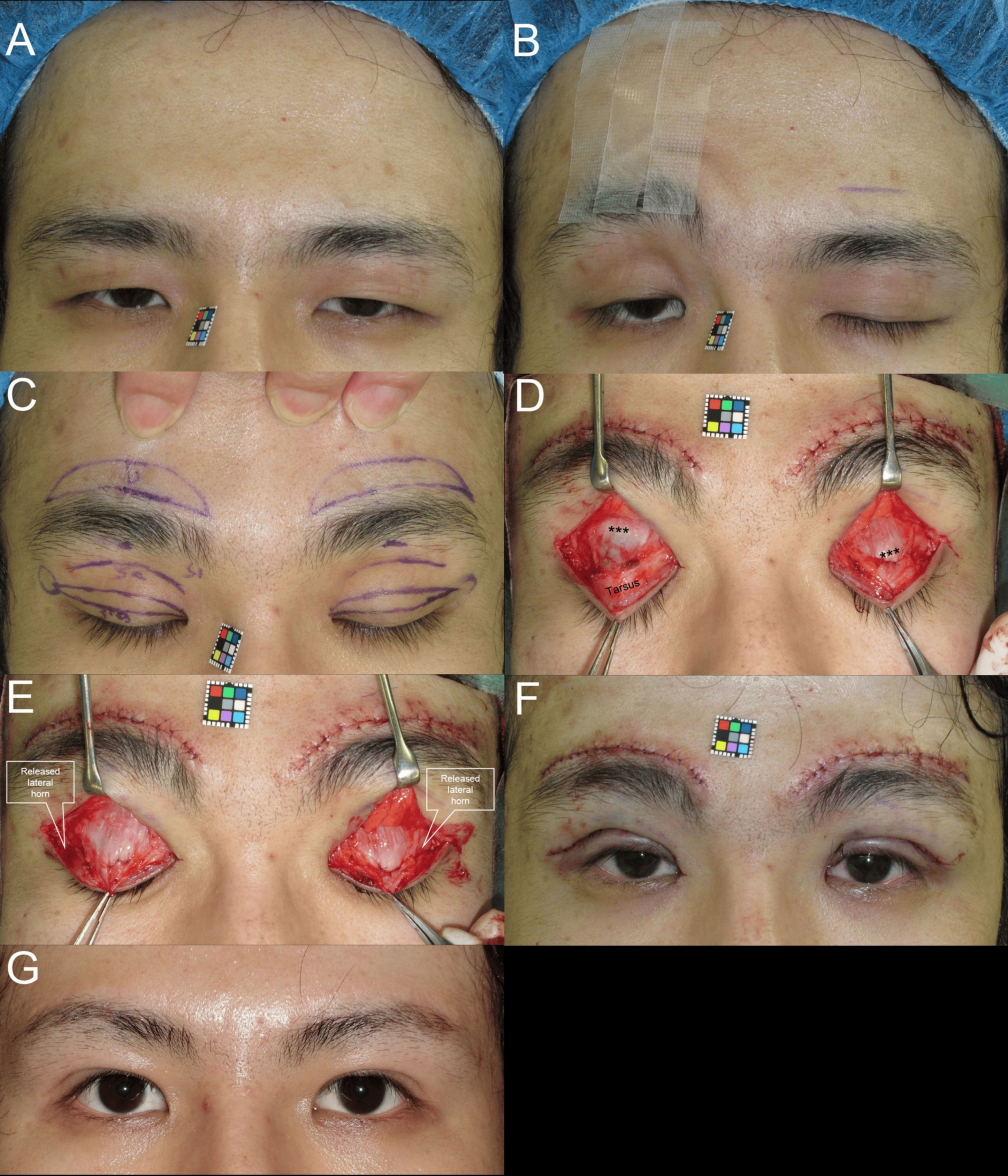
Case 1. Clinical photographs and surgical findings (A) Primary gaze demonstrating increased contractions of the orbital OOM. (B) Unilateral right eyebrow lifting with tape. (C) Preoperative surgical markings for skin excision above the eyebrows and along the superior palpebral creases. (D) Intraoperative view following bilateral eyebrow skin closure and reinsertion of the left aponeurosis using a 6-0 nonabsorbable suture. Upward traction on the lid margins reveals severe disinsertion of the right aponeurosis from the tarsus with significant slippage. Asterisks (***) indicate the distal margins of the aponeuroses. (E) Bilateral aponeuroses reinserted into the tarsi. The lateral horns were released to facilitate physiologic stretching of the STM during eyelid opening. (F) Primary gaze immediately postoperatively. (G) Primary gaze at the 18-month postoperative follow-up. OOM: orbicularis oculi muscle; STM: superior tarsal muscle

A 29-year-old man presented with severe levator aponeurosis disinsertion caused by habitual eyelid rubbing. To maintain eyelid opening, he required increased voluntary contractions and microsaccades of FTFs in the LPSM and GLSRM, which significantly stretched the STM mechanoreceptors and secondarily increased orbital OOM contractions (Figure 2A) [9]. Clinically, he exhibited limb dystonia. He complained of heightened contractions in his limb flexor muscles, which severely impaired manual dexterity and ambulation. A gait test revealed marked dystonic contractions of the hip and knee flexors (Video 1). A finger-to-nose test (Video 2) demonstrated dystonic contractions of the shoulder and elbow flexors, including pronators, whereas the limb extensors were unaffected.

**Video 1:** Case 1. Preoperative gait test.

**Video 2:** Case 1. Preoperative finger-to-nose test without taping

To reduce STM mechanoreceptor stretching during eyelid opening, unilateral eyebrow lifting was performed using tape to a degree that prevented complete eyelid closure during light OOM contraction (Figure 2B). Interestingly, this intervention immediately resolved the dystonic spasms in the ipsilateral (right) arm during the finger-to-nose test, whereas the contralateral (left) arm remained completely dystonic and unaffected (Videos 3, 4), demonstrating a somatotopic lateralization of the proprioceptive reflex.

**Video 3:** Case 1. Finger-to-nose test during taping of the right eyebrow

**Video 4:** Case 1. Finger-to-nose test during taping of the left eyebrow

The patient subsequently underwent bilateral surgical correction, consisting of eyebrow lifting via fusiform skin excision, reduction of redundant upper eyelid skin around the superior palpebral crease (Figure 2C), and reinsertion of the severely disinserted aponeuroses into the tarsi (Figures 2D, 2E) [13]. Immediately postoperatively, the dystonic finger-to-nose test normalized (Video 5). At the one-week follow-up, his gait dystonia was fully resolved (Video 6). At 18 months postoperatively, the limb dystonia remained completely cured (Figure 2G, Video 7).

**Video 5:** Case 1. Immediate postoperative finger-to-nose test

**Video 6:** Case 1. Gait test 1 week postoperatively.

**Video 7:** Case 1. Finger-to-nose test 18 months postoperatively

Case 2

**Figure 3:**
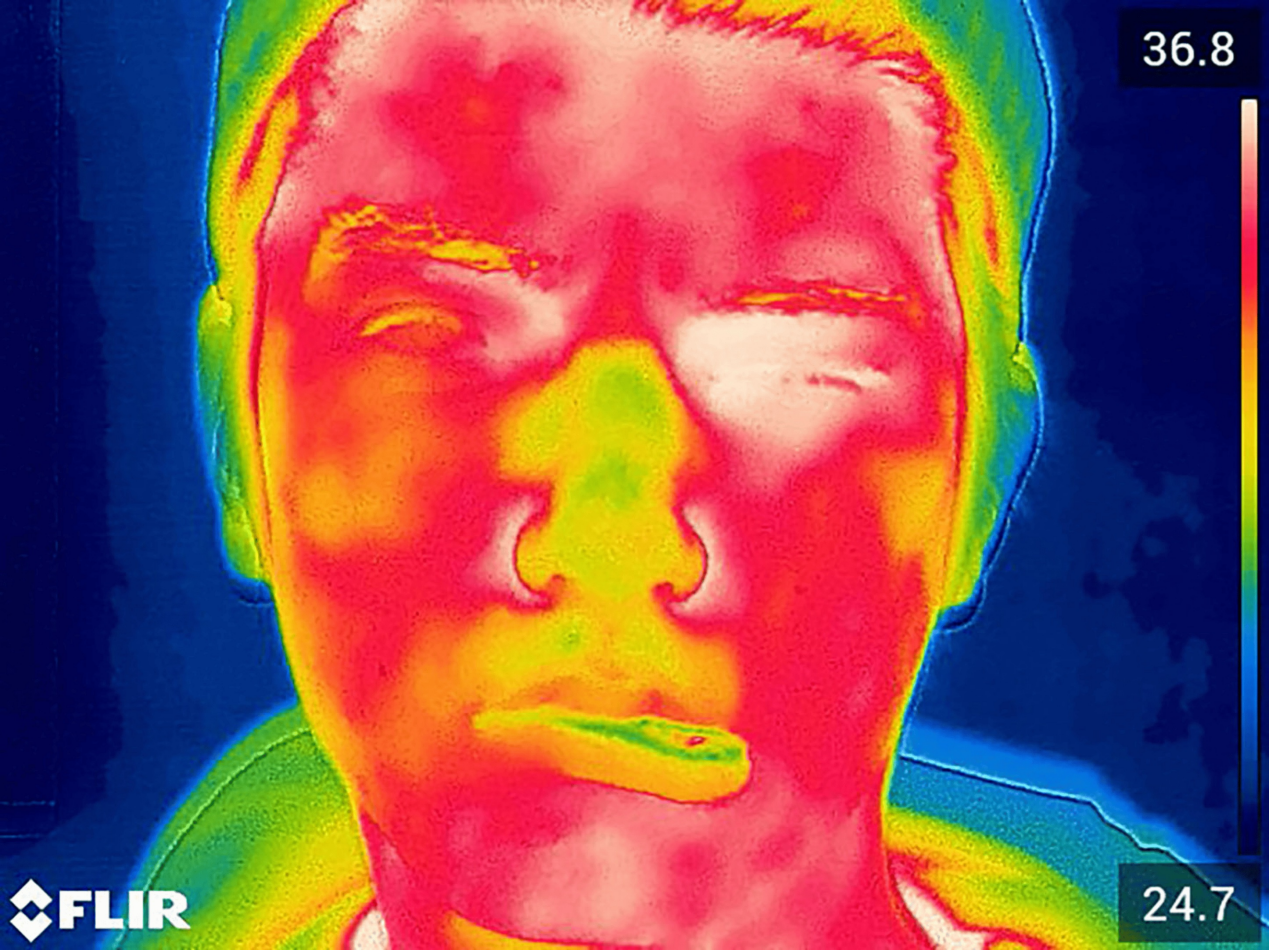
Case 2. Facial thermographic analysis Thermography reveals an elevated temperature in the left palpebral OOM and depressor labii inferioris muscle, reflecting continuous reflex contractions of slow-twitch fibers within these muscle groups.

A 38-year-old man presented with multifocal dystonia characterized by involuntary facial grimacing, tongue thrusting, jaw opening, and neck rotation (Video 8), accompanied by left-predominant limb flexion dystonia during finger-to-nose (Video 9) and gait (Video 10) tests. His symptoms developed following transconjunctival orbital fat decompression surgery for lower eyelid bags. The surgery had iatrogenically sensitized putative mechanoreceptors in the left ITM, leading to increased reflex contractions of the left palpebral OOM [12]. Thermography revealed an elevated temperature in the left palpebral OOM (Figure 3). Because upper eyelid opening and upgaze counteracted the hypercontracted palpebral OOM, these movements significantly exacerbated STM mechanoreceptor stretching and worsened the dystonic spasms; conversely, downgaze reduced STM mechanoreceptor stretching and relieved the symptoms (Video 8). Mechanically pulling down the left lower eyelid relieved the dystonia, whereas raising it worsened the contractions (Video 11).

**Video 8:** Case 2. Left-predominant multi-focal dystonia Spasms of the face, tongue, jaw, neck, and upper limb flexors are exacerbated by upgaze and relieved by downgaze.

**Video 9:** Case 2. Dystonic finger-to-nose test

**Video 10:** Case 2. The gait test demonstrates dystonic spasms of the leg flexors as well as internal rotators

**Video 11:** Case 2. Response to mechanical manipulation of the left lower eyelid (alleviation via downward traction; exacerbation via elevation)

Simultaneous taping to lift the left eyebrow and pull down the left cheek reduced mechanoreceptor stretching in both the STM and ITM, which significantly alleviated the left-sided craniocervical spasms (Video 12) and normalized limb function during finger-to-nose (Video 13) and gait (Video 14) tests. Removing the eyebrow tape while maintaining the cheek tape continued to suppress the dystonic movements (Video 12). However, manually elevating the lower eyelid margin (Video 11) or removing the cheek tape (Video 15) immediately provoked a recurrence of the dystonia. Notably, high-frequency vibration stimuli applied to the first (ophthalmic) and second (maxillary) branches of the trigeminal nerve, presumably masking the abnormal proprioceptive input from STM stretching, completely alleviated the facial grimacing, tongue thrusting, jaw opening, and neck rotation (Video 16).

**Video 12:** Case 2. Amelioration of craniocervical dystonia via concurrent left eyebrow elevation and left cheek depression with tape, which persists after removing only the eyebrow tape

**Video 13:** Case 2. Improvement in the finger-to-nose test during taping

**Video 14:** Case 2. Improvement in gait during taping

**Video 15:** Case 2. Reappearance of dystonic spasms upon removal of facial tapes

**Video 16:** Case 2. Complete resolution of craniocervical dystonia during high-frequency vibration stimuli to the ophthalmic and maxillary branches of the trigeminal nerve

Table 1 provides a clinical summary of the two patients, interventions, and outcomes.

**Table 1:** Case summary

Parameter	Case 1	Case 2
Age / Sex	29-year-old male	38-year-old male
Etiology	Habitual eyelid rubbing leading to bilateral aponeurosis disinsertion	Iatrogenic sensitization of left ITM via transconjunctival orbital fat surgery
Baseline Clinical Presentation	Task-specific limb flexion dystonia (gait/finger-to-nose)	Left-predominant multifocal dystonia (facial grimacing, tongue thrusting, jaw opening, neck rotation, limb flexion)
Differential Workup (Excluded)	Primary torsion dystonia, Wilson’s disease, drug-induced dystonia (via neuro-consultation wth MRI, labs, history)	Structural brain lesions, metabolic or tardive dystonias (via neuro-consultation)
Response to Taping Test	Immediate resolution of ipsilateral limb dystonia; contralateral limb remained dystonic	Significant alleviation of left-sided craniocervical and limb spasms
Response to Trigeminal Vibration	Not tested	Complete temporary resolution of craniocervical and tongue spasms (V1/V2 stimulation)
Definitive Treatment	Bilateral surgical eyebrow lift and levator aponeurosis reinsertion	Conservative facial taping (long-term surgical desensitization planned)
Follow-up & Outcome	Complete resolution of limb dystonia was maintained at 18 months	Immediate relief is maintained during active taping/vibration; symptoms recur upon removal

## Discussion

In both cases, the observed limb dystonia was highly task-specific, manifesting predominantly during the finger-to-nose and gait tests. Notably, the limb flexor muscles exhibited greater contraction than the extensor muscles during these tasks. In the finger-to-nose test (Videos 2, 9), the movement of the finger toward the nose provoked dystonic spasms, whereas removing the finger did not. Similarly, forward gait was impaired by increased dystonic contractions of limb flexor muscles and concurrent reciprocal inhibition of limb extensors (Videos 1, 10) (Figure 1), while backward gait remained relatively unaffected (Video 10).

Reducing the mechanical stretch on the STM mechanoreceptors, either temporarily via taped eyebrow elevation (Figure 2B; Videos 3, 4, 12-15) or permanently via corrective surgery (Figures 2C-2F), immediately alleviated these symptoms. This intervention decreased the involuntary contractions of the ipsilateral limb flexors and restored extensor activity (Videos 3-7, 13, 14), presumably by downregulating the hyperactivated rostral LC. Because the LC exerts a tonic excitatory influence on limb flexors and a tonic inhibitory influence on limb extensors [14-18], we propose a working hypothesis that this specific limb dystonia is driven by an imbalance of spinal motor signaling (increased flexor excitation and extensor inhibition) secondary to STM mechanoreceptor overstimulation via the MTN and rostal LC (Figure 1). This proposed mechanism may interact with the monoaminergic (noradrenergic) descending control system from the brainstem to the spinal cord, as well as indirect motor regulation mechanisms mediated via pathways like the reticulospinal tract [14-18]. While these observations are clinically striking, they remain bounded by the descriptive nature of a two-case report, and definitive electrophysiological confirmation, such as quantified electromyography or functional neuroimaging, is warranted to establish causality.

We previously reported that when voluntary contractions and microsaccades of FTFs in the LPSM and GLSRM stretch STM mechanoreceptors, STFs in the facial expression muscles reflexively contract via the MTN-LC-brainstem axis (Figure 1) [9]. In the current cases, the heightened reflex contractions of STFs in the palpebral OOM [12] structurally opposed upper eyelid opening. This muscular resistance generated profound, continuous stretching of the STM mechanoreceptors, reflexively precipitating dystonic spasms in the facial expression and grimacing muscles (Figure 1, Video 8) [9].

Furthermore, since the LC projects to the hypoglossal nucleus [14,15], abnormal STM mechanoreceptor stretching may reflexively induce dystonic contractions of STFs in the genioglossus muscle, resulting in tongue thrusting. Similarly, because the LC sends projections to the trigeminal motor nucleus [14,15], excessive STM input may drive dystonic STF contractions in the jaw-opening muscles (geniohyoid, mylohyoid, anterior belly of the digastric, and lateral pterygoid).

Because the LPSM originates from the sphenoid bone and inserts into the upper tarsus with a lateral deviation of approximately 23 degrees, lateral gaze inherently induces asymmetric stretching of bilateral STM mechanoreceptors. Mechanoreceptors on the ipsilateral side of the lateral gaze undergo greater stretch, increasing reflex STF contractions in the LPSM and frontalis muscle to elevate the eyelid and eyebrow; conversely, the contralateral side experiences less stretch, causing slight ptosis and eyebrow drooping. When horizontal gaze exceeds physiological limits without head movement, the head spontaneously rotates via involuntary contraction of the contralateral sternocleidomastoid (SCM) muscle and the ipsilateral levator scapulae muscle. This suggests that enhanced asymmetric stretching of STM mechanoreceptors on the side of the lateral gaze reflexively recruits the contralateral SCM muscle [10]. In Case 2, the unilateral, iatrogenic hyper-contraction of the left palpebral OOM created a chronic asymmetric stretch of the left STM and ITM mechanoreceptors during primary gaze [12]. This likely provoked reflex contractions of the contralateral SCM muscle and/or ipsilateral levator scapulae muscle, resulting in involuntary neck rotation (Videos 8, 15, 16) [10]. 

On the dystonic side, high-frequency focal vibration applied via a smartphone-based motor component to the skin above the lateral eyebrow (innervated by the lacrimal nerve of the trigeminal ophthalmic branch (V1)) and over the infraorbital foramen (innervated by the maxillary branch (V2)) successfully aborted the dystonic spasms (Video 16). Because STM mechanoreceptors are innervated by the medial branch of the lacrimal nerve [3,5], V1 vibration stimuli likely masked the abnormal proprioceptive input from the stretched STM, suppressing craniocervical and tongue spasms. Similarly, since putative ITM mechanoreceptors are innervated by the V2 branch, infraorbital vibration may mask ITM proprioception, thereby reducing reflex contractions of the palpebral OOM [12]. This sensory gating mechanism provides a plausible explanation for the "hanger reflex": gentle pressure applied by a wire hanger above the lateral eyebrow evokes ipsilateral trigeminal proprioception, reflexively contracting the contralateral SCM and causing the head to rotate toward the side of the stimulus.

Given that Meige syndrome is clinically characterized by dystonic spasms of the facial expression, tongue, jaw, and neck muscles [19,20], pathological mechanoreceptor overstimulation in both the STM and ITM may serve as a critical upstream trigger that hyperactivates brainstem motor centers, presenting as a Meige-syndrome phenotype.

Patients with tarsal disinsertion of the levator aponeurosis or inferior tarsal retractor must excessively contract the FTFs of the LPSM and GLSRM to achieve adequate eyelid opening, which hyper-activates the rostral LC via chronic STM stretching. Concurrently, heightened compensatory activity of the GLIRM FTFs stretches ITM mechanoreceptors, increasing reflex contractions of the palpebral OOM STFs [12]. This internal resistance further exacerbates upper eyelid opening resistance and subsequent STM stretching, potentially culminating in a combination of limb dystonia and Meige syndrome [19,20]. Consequently, mechanical interventions (eyebrow elevation and cheek depression via taping) or sensory trick-like vibration stimuli to the V1 and V2 pathways may serve as highly useful tools for the differential diagnosis of limb dystonia and Meige syndrome. Ultimately, surgical desensitization or readjustment of the STM and ITM mechanoreceptor stretch parameters represents a promising, novel therapeutic strategy for these debilitating movement disorders.

The rostral LC, modulated by STM mechanoreceptor inputs during vertical eye movements, projects widely to the forebrain and limbic systems, regions deeply intertwined with neuropsychiatric and affective disorders. During the horizontal, rhythmic eye movements utilized in Eye Movement Desensitization and Reprocessing (EMDR) therapy, as well as rapid eye movement (REM) sleep, the undulating, alternating stretch of bilateral STM mechanoreceptors likely induces a transient, cyclic modulation of the rostral LC, distinct from the continuous tone maintained during a static primary gaze. This dynamic kinetic modulation may facilitate the desensitization and reprocessing of hyperactive noradrenergic networks, thereby alleviating psychological distress. Consequently, non-invasive interventions, such as facial taping and V1/V2 trigeminal focal vibration, or surgical adjustment of the STM/ITM mechanoreceptor architecture, may hold novel therapeutic potential for psychiatric conditions rooted in LC dysregulation.

## Conclusions

Significant, pathological stretching of mechanoreceptors in the STM is proposed to hyperactivate the rostral LC, which may exert a tonic excitatory influence on limb flexor motor neurons and a reciprocal inhibitory influence on limb extensor pathways, contributing to task-specific limb dystonia. Concurrently, this proprioceptive overstimulation is hypothesized to drive brainstem motor centers to reflexively induce dystonic contractions of the facial grimacing, tongue thrusting, jaw opening, and neck rotation muscles, closely mimicking the clinical phenotype of Meige syndrome. While the immediate symptom resolution via mechanical taping and trigeminal vibration in these two cases offers compelling preliminary evidence, these findings must be interpreted as hypothesis-generating. Future prospective, controlled studies with standardized movement disorder scoring scales and electrophysiological validation are required to formally establish this ocular-proprioceptive pathway in the broader pathophysiology of craniocervical and peripheral dystonias.
